# Metabolic–Nutritional Associations with Depression in Elderly Chronic Kidney Disease Patients: Hemodialysis Versus Non-Dialysis Populations

**DOI:** 10.3390/metabo15110710

**Published:** 2025-10-30

**Authors:** Sedat Ozdemir, Aynur Ekren Cakici, İbrahim Bilir

**Affiliations:** 1 Department of Internal Medicine, School of Medicine, Gaziantep Islam Science and Technology University, 27010 Gaziantep, Turkey; 2 Department of Dialysis, Vocational School, Hasan Kalyoncu University, 27010 Gaziantep, Turkey; aynur.ekren@hku.edu.tr; 3 Department of First and Emergency Aid, Hasan Kalyoncu University, 27470 Gaziantep, Turkey; ibrahim.bilir@hku.edu.tr

**Keywords:** chronic kidney disease, geriatric nephrology, hemodialysis, depression, nutritional status, integrated care, quality of life

## Abstract

**Background/Objectives:** This study aimed to evaluate the interrelationship between depression and nutritional–metabolic status in geriatric patients with chronic kidney disease (CKD), and to identify the contributing clinical and sociodemographic factors, with a particular focus on differences between patients receiving and not receiving hemodialysis treatment. **Methods:** This cross-sectional descriptive study was conducted between September 2023 and September 2024 in Gaziantep, Turkey. A total of 152 CKD patients aged 65 years and older were included, with 78 receiving hemodialysis and 74 managed without dialysis. Nutritional status was assessed using the Mini Nutritional Assessment—Short Form (MNA-SF), and depression levels were measured using the Geriatric Depression Scale—Short Form (GDS-SF). Laboratory parameters such as hemoglobin and serum albumin were also recorded. **Results:** A total of 152 geriatric CKD patients were included, with 78 (51.3%) undergoing hemodialysis and 74 (48.7%) not receiving dialysis. The non-dialysis group had significantly higher age (77.07 ± 8.61 vs. 70.13 ± 7.76 years, *p* = 0.001) and BMI (28.44 ± 4.70 vs. 25.18 ± 4.75, *p* = 0.001). Serum albumin levels were lower in non-dialysis patients (2.53 ± 0.67 vs. 3.50 ± 0.465 g/dL, *p* < 0.001), while hemoglobin (12.44 ± 2.03 vs. 11.16 ± 1.92 g/dL, *p* = 0.001) and ALT levels (17.16 ± 13.06 vs. 8.53 ± 4.31 U/L, *p* = 0.001) were higher. Malnutrition was more frequent among non-dialysis patients (20.3% vs. 9.0%, *p* = 0.033). Although depression was more common in this group, the difference was not statistically significant (*p* = 0.091). A moderate negative correlation was observed between MNA-SF and GDS-SF scores (r = −0.426, *p* < 0.01). Serum albumin positively correlated with MNA-SF (r = 0.374, *p* < 0.01) and negatively with GDS-SF (r = −0.323, *p* < 0.01). **Conclusions:** Malnutrition was more frequent among elderly CKD patients not receiving hemodialysis, whereas depression prevalence did not differ significantly between groups. A significant correlation between nutritional status (MNA-SF, serum albumin) and depressive symptoms (GDS-SF) was observed in the overall CKD cohort, underscoring the close interplay between metabolic and psychological health in this population. These results highlight the need for routine screening and integrated management of both nutrition and mental health in elderly CKD patients, and future longitudinal studies are warranted to determine causal pathways and predictive value.

## 1. Introduction

Aging is an irreversible process characterized by progressive biological, physiological, psychological, and social changes over the lifespan [1]. As global demographics shift, the proportion of the geriatric population is steadily increasing. In 2020, older adults accounted for 9.3% of the global population, rising to 10.3% by 2024, with projections estimating an increase to 16% by 2050 and 20.7% by 2074 [2,3]. In Turkey, the elderly population rose from 9.5% in 2020 to 10.6% in 2024 [4].

With aging, the prevalence of chronic diseases such as cardiovascular disease, osteoarthritis, and Alzheimer’s disease increases significantly, leading to prolonged morbidity and functional dependency [5,6,7,8,9]. In the absence of underlying pathology, renal function generally remains stable in older individuals. However, the glomerular filtration rate (GFR) decreases by approximately 10% per decade after the age of 40, accompanied by reductions in renal plasma flow, creatinine clearance, muscle mass, and urinary creatinine excretion [10,11,12,13,14,15,16]. These changes necessitate careful adjustment of drug dosages in the elderly.

Chronic kidney disease (CKD) is a progressive, irreversible, and multisystemic disorder with increasing prevalence due to rising rates of diabetes and hypertension, as well as population aging [6,14,16,17,18,19,20,21]. Recent meta-analytic evidence from 65 studies suggests that the pooled prevalence of clinical depression among CKD patients globally is 26.5% (95% CI: 23.1–30.1%) [22]. In the United States, estimates in CKD populations range from approximately 10% to 20% depending on measurement method and disease stage [23]. Although there is no large nationwide CKD-depression survey in Turkey, the 2022 Turkish Society of Nephrology registry reports a prevalence of 1016. 2 ESRD patients per million, underscoring the growing burden of renal disease in the country [24].

Early-stage CKD is often asymptomatic, and diabetes mellitus (DM) and hypertension (HT) are the leading causes [25,26]. Symptoms typically emerge when GFR drops below 35–50 mL/min/1.73 m^2^, with nocturia being one of the earliest clinical signs. As GFR further declines to 20–25 mL/min/1.73 m^2^, uremic symptoms become evident, often accompanied by cardiovascular complications, dyslipidemia, bone-mineral disorders, endocrine abnormalities, and electrolyte imbalances [25,26].

Advances in dialysis technologies and care protocols have extended the life expectancy of CKD patients, yet new challenges have emerged, particularly among elderly dialysis recipients. These patients are more sensitive to rapid volume shifts, show reduced treatment adherence, and experience higher rates of malnutrition, hypoalbuminemia, inflammation, and cardiovascular comorbidities compared to their younger counterparts [27,28].

Malnutrition is defined as a deficiency in energy and nutrient intake or increased metabolic demands, leading to loss of lean body mass and functional decline [29]. Its prevalence in the elderly varies between 20 and 30% and is particularly high in hospitalized patients, those undergoing renal replacement therapy, or living in long-term care institutions [30,31,32]. Multiple contributing factors include physiological impairments (e.g., dysphagia, sensory loss), chronic illnesses, psychological conditions (e.g., depression, dementia), and social determinants such as isolation and poverty [33,34].

Serum albumin is widely used as an indicator of nutritional status. However, levels may be influenced by various factors, including inflammation. Low serum albumin is associated with increased mortality, sarcopenia, and poor functional outcomes in older adults [35,36,37,38]. Among dialysis patients, factors such as poor dietary compliance, taste alterations due to uremia, anorexic effects of dialysate, and psychological burden also contribute to impaired nutritional intake [33,39,40].

Depression is the most common psychiatric condition observed in CKD patients, with prevalence estimates ranging from 10% to 20%, exceeding rates seen in other chronic conditions such as diabetes and heart failure [41,42]. In older adults, depression may stem from both biological and psychosocial causes, including chronic disease burden, loss of independence, bereavement, and social isolation [43]. Geriatric depression can impair quality of life, exacerbate malnutrition, increase mortality risk, and complicate the management of comorbid conditions [44].

Although depression and malnutrition have been studied separately in CKD, few studies have directly compared geriatric dialysis and non-dialysis populations using standardized tools such as the Mini Nutritional Assessment—Short Form (MNA-SF) and Geriatric Depression Scale—Short Form (GDS-SF) simultaneously [45,46,47]. Our study addressed this gap. Given the bidirectional association between nutritional status and psychological health, we aimed to assess the relationship between depression and malnutrition in elderly patients with chronic kidney disease and to identify the contributing sociodemographic and clinical factors. By clarifying these associations, the study holds broader significance for developing integrated screening approaches that may improve early detection, guide holistic management, and ultimately enhance quality of life in this vulnerable population.

## 2. Materials and Methods

### 2.1. Study Design

The study population consisted of patients with a documented diagnosis of CKD established by a nephrologist, based on KDIGO criteria (eGFR < 60 mL/min/1.73 m^2^ for at least 3 months and/or supportive clinical/laboratory findings), who were either receiving home healthcare services or undergoing hemodialysis treatment at three private dialysis centers in Gaziantep, Turkey, between 1 September 2023, and 30 January 2024. Among the 74 non-dialysis patients, the majority were in CKD stage 3–4, with a smaller proportion in stage 5 but not yet on dialysis.

No specific sampling method was employed; instead, all patients who met the inclusion criteria and voluntarily agreed to participate were enrolled. Patients who met the inclusion criteria and voluntarily agreed to participate were enrolled. Inclusion criteria were: (1) age ≥ 65 years; (2) confirmed diagnosis of CKD based on clinical and/or laboratory findings; (3) ability to participate in face-to-face interviews; and (4) provision of written informed consent. Exclusion criteria were: severe cognitive impairment, acute medical conditions requiring hospitalization, active malignancy, acute inflammatory diseases, or unwillingness to participate. Patients with common and stable comorbidities (such as hypertension, diabetes, or cardiovascular disease) were not excluded to better reflect the real-world CKD population.

### 2.2. Data Collection Tools

Data were collected through face-to-face interviews using structured data collection forms. The process took approximately 20 min per participant. The following instruments were used:

- **Personal Information Form:** Developed by the researchers to collect sociodemographic and clinical data, including age, gender, marital status, education level, occupation, disease duration, smoking and alcohol use, and body mass index (BMI). BMI was categorized according to the World Health Organization (WHO) criteria: under-weight (<18.5 kg/m^2^), normal weight (18.5–24.9 kg/m^2^), overweight (25.0–29.9 kg/m^2^), and obesity (≥30.0 kg/m^2^) [48].
- **Mini Nutritional Assessment—Short Form (MNA-SF):** This validated tool evaluates nutritional status using six parameters: recent weight loss, appetite, mobility, psychological stress, neuropsychological issues, and BMI. We selected the MNA-SF because it is specifically designed for geriatric populations and has been extensively validated as a practical and reliable screening instrument. The Turkish version of the MNA-SF has also been validated and shown to be reliable in elderly populations [49]. Although biomarker panels (e.g., prealbumin, transferrin, CRP) may provide additional objective insights, they are less feasible in routine geriatric care due to cost and accessibility. To strengthen our assessment, we also incorporated serum albumin, hemoglobin, and ALT values as complementary laboratory markers [50].
- **Geriatric Depression Scale—Short Form (GDS-SF)**: Developed to screen for depressive symptoms in older adults, this 15-item questionnaire includes yes/no responses. Each item is scored 0 or 1, with total scores classified as follows: 0–4 = normal, 5–8 = mild depression, 9–11 = moderate depression, and 12–15 = severe depression [51]. The Turkish version of the GDS-SF has been validated, demonstrating good reliability and internal consistency in elderly patients [52].
- **EQ-5D General Health Questionnaire**: This tool was included to evaluate participants’ perceived quality of life across five domains: mobility, self-care, usual activities, pain/discomfort, and anxiety/depression [53].

**Figure 1:**
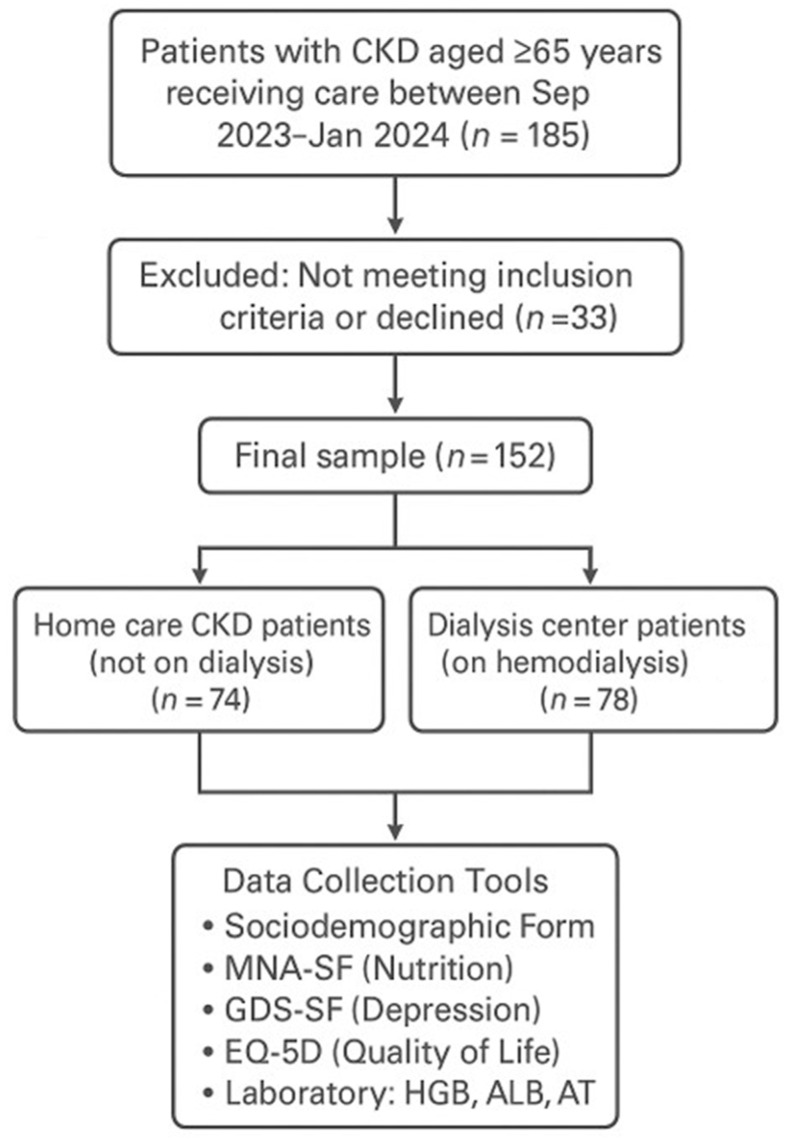
Flowchart of the study.

Laboratory data, including hemoglobin (HGB), serum albumin (ALB), and alanine aminotransferase (ALT) levels, were obtained from patient records during clinical visits. Hemoglobin levels were determined using an automated hematology analyzer (Sysmex XN-1000, Sysmex Corporation, Kobe, Japan). Serum albumin concentrations were measured by the bromocresol green (BCG) colorimetric method (Roche Cobas c702, Roche Diagnostics, Mannheim, Germany). ALT activity was assessed using an enzymatic kinetic method on an automated chemistry analyzer (Roche Cobas c702, Roche Diagnostics, Mannheim, Germany) (Figure 1).

### 2.3. Statistical Analysis

All statistical analyses were performed using IBM SPSS Statistics version 27.0 (IBM Corp., Armonk, NY, USA). Continuous variables were expressed as mean ± SD for normally distributed data and as median (IQR) for non-normally distributed data. Categorical variables were presented as frequencies and percentages. The normality of data distribution was assessed using the Kolmogorov–Smirnov test for the overall sample (n > 50). For smaller subgroup analyses, the Shapiro–Wilk test was additionally applied. This combined approach ensured appropriate evaluation of distributional assumptions depending on sample size. For group comparisons, the Independent Samples *t*-test was used for normally distributed variables, and the Mann–Whitney U test for non-normally distributed variables. Outliers were assessed by boxplot and z-score inspection; cases with z-score > ±3 were excluded from analyses. Correlation analysis was performed using Pearson’s test for normally distributed variables and Spearman’s rank correlation for non-normally distributed variables. A *p*-value < 0.05 was considered statistically significant.

## 3. Results

Of the 152 geriatric patients with chronic kidney disease included in the study, 78 (51.3%) were undergoing hemodialysis, while 74 (48.7%) were managed without dialysis. Gender distribution was similar between the groups (female: 51.3% in hemodialysis vs. 56.8% in non-hemodialysis). Most patients were married in both groups (74.4% vs. 74.3%). Educational attainment was generally low, with illiteracy more common among non-hemodialysis patients (59.5% vs. 35.9%). Smoking and alcohol use showed no significant differences between groups (smoking: 25.6% vs. 23.0%; alcohol use: 7.7% vs. 4.1%). Regarding nutritional status based on BMI, obesity was more frequent in the non-hemodialysis group (29.7% vs. 19.2%), while overweight status was similar across groups. Among hemodialysis patients, 39.7% had been on treatment for six years or more, and the majority (75.6%) underwent dialysis three times per week (Table 1).

When comparing physical and laboratory parameters between hemodialysis and non-hemodialysis patients, several statistically significant differences were observed. Non-dialysis patients were significantly older than those receiving hemodialysis (77.07 ± 8.61 vs. 70.13 ± 7.76 years, *p* = 0.001). They also had a higher body mass index (BMI) (28.44 ± 4.70 vs. 25.18 ± 4.75, *p* = 0.001) and greater body weight (74.95 ± 11.34 vs. 69.83 ± 12.05 kg, *p* = 0.008), although their height was slightly lower (164.16 ± 6.52 vs. 166.83 ± 7.60 cm, *p* = 0.021). Regarding laboratory values, non-dialysis patients had significantly lower serum albumin levels (2.53 ± 0.67 vs. 3.50 ± 0.465 g/dL, *p* < 0.001). In contrast, their hemoglobin (12.44 ± 2.03 vs. 11.16 ± 1.92 g/dL, *p* = 0.001) and ALT levels (17.16 ± 13.06 vs. 8.53 ± 4.31 U/L, *p* = 0.001) were significantly higher than those of hemodialysis patients (Table 2).

Analysis of nutritional and depression scores revealed differences between the hemodialysis and non-hemodialysis groups. According to the MNA-SF, the prevalence of malnutrition was higher among non-dialysis patients (20.3%) than hemodialysis patients (9.0%), while the proportion at risk was similar (28.3% vs. 25.6%). A greater percentage of hemodialysis patients had normal nutritional status (65.4% vs. 51.4%) (*p* = 0.033). Regarding depression levels based on the GDS-SF, no overall statistically significant difference was found between groups (*p* = 0.091). However, mild depression was nearly identical in both groups (23.0% vs. 21.8%), while moderate depression was more frequent in the non-dialysis group (28.4% vs. 9.0%). Severe depression occurred less commonly, but was observed more in hemodialysis patients (6.4% vs. 1.4%) (Table 3).

**Figure 2:**
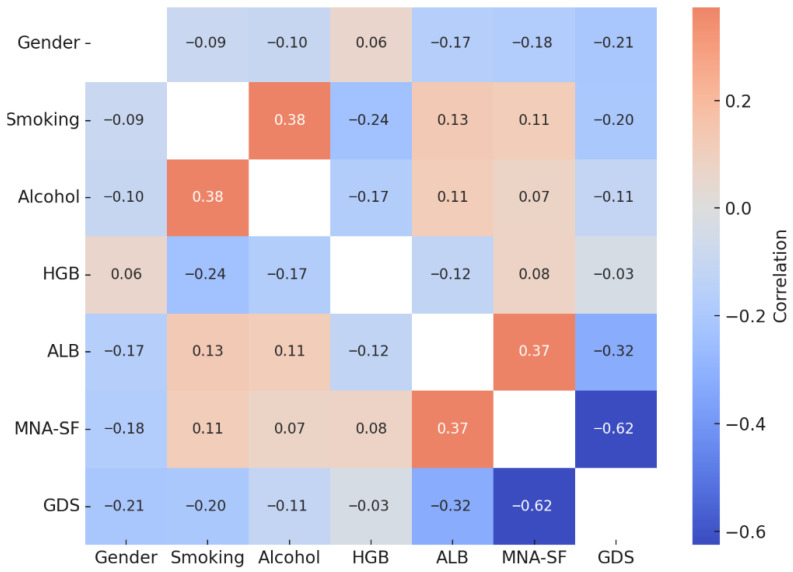
Heatmap of correlations between sociodemographic, laboratory, nutritional, and psychological variables in elderly CKD patients (entire cohort, n = 152). HGB, hemoglobin; ALB, serum albumin; MNA-SF, Mini Nutritional Assessment–Short Form; GDS-SF, Geriatric Depression Scale–Short Form; BMI, body mass index.

Correlation analysis revealed significant associations between nutritional, psychological, and laboratory parameters in the study population. There was a moderate negative correlation between MNA-SF (nutritional status) and GDS-SF scores (depression) (r = −0.625, *p* < 0.01), indicating that as nutritional status worsens, depressive symptoms increase. Serum albumin levels showed a positive correlation with MNA-SF scores (r = 0.374, *p* < 0.01) and a negative correlation with GDS-SF scores (r = −0.323, *p* < 0.01), suggesting that higher albumin levels are associated with better nutritional and psychological status. In addition, hemoglobin levels were inversely correlated with smoking (r = −0.236, *p* < 0.01) and alcohol use (r = −0.173, *p* < 0.05). GDS-SF scores were also significantly and negatively correlated with gender (r = −0.209, *p* < 0.01) and smoking status (r = −0.199, *p* < 0.05), indicating that male gender and smoking may be linked to higher depressive symptoms (Table 4, Figure 2).

## 4. Discussion

This study compared the socio-demographic, clinical, nutritional, and psychological profiles of elderly CKD patients undergoing hemodialysis and those managed without dialysis. The results showed that while both groups shared many baseline characteristics, non-dialysis patients were significantly older, had higher BMI, and displayed poorer nutritional markers, particularly lower serum albumin. Malnutrition was more prevalent in non-dialysis patients, likely due to older age, comorbidities, and the absence of structured nutritional monitoring inherent to dialysis care. Pathophysiologically, factors such as sarcopenia, chronic inflammation, and protein-energy wasting may have contributed to lower albumin levels. By contrast, depression scores did not differ significantly between the groups, which suggests that psychological burden is a common feature of CKD regardless of dialysis status. These findings underscore the need to interpret depression and malnutrition as interrelated but not exclusively dialysis-dependent phenomena.

Although the non-dialysis group was significantly older, the literature suggests that age alone does not fully account for the nutritional disparities observed between dialysis and non-dialysis CKD patients. Bellanti et al. and Moldovan et al. reported that while advancing age is associated with mild declines in albumin, dialysis-specific factors such as inflammation control, metabolic acidosis management, and dietary counseling have a stronger influence on malnutrition risk [29,32]. Sahathevan et al. emphasized that routine nutritional monitoring during hemodialysis can mitigate age-related nutritional deterioration [33]. Therefore, although age may have contributed partially to lower albumin and higher malnutrition prevalence in the non-dialysis group, the persistence of significant inter-group differences after accounting for these factors in comparable studies suggests that dialysis-related mechanisms likely play a more direct role.

In our sample, 20.3% of non-dialysis patients had malnutrition compared to 9.0% of dialysis patients. This suggests that regular hemodialysis treatment may contribute to improved nutritional regulation. Comparable trends have been reported in the literature, where malnutrition and hypoalbuminemia were frequently observed in elderly CKD patients, especially in advanced stages and those not receiving dialysis [29,31,33].

Separately, regarding psychological outcomes, previous studies have consistently shown that depression is highly prevalent in CKD patients. For example, Wang et al. reported a depression prevalence of 23% among pre-dialysis elderly CKD patients, while de Alencar et al. found a prevalence of 22.5% in hemodialysis patients aged 60 years and older [46,47]. Chu et al. observed a much higher prevalence of 65.3% in a younger hemodialysis cohort, and Liu et al. reported mild, moderate, and severe depression rates of 23%, 15%, and 13%, respectively, among dialysis patients [2,54]. These findings confirm that depression is highly prevalent in CKD, regardless of dialysis status [41,42,43].

Interestingly, hemodialysis patients in our cohort demonstrated relatively preserved albumin levels despite lower BMI and the general expectation of higher malnutrition prevalence. This paradox may be explained by the regular laboratory monitoring and nutritional counseling inherent to dialysis care, as well as possible selection bias, since non-dialysis patients were recruited from home care services and may represent a frailer subgroup. At the same time, lower albumin was associated with higher depressive symptom scores in our cohort. Prior research supports the association between malnutrition and depression: Guenzani et al. found a link between malnutrition, inflammation, depression, and cognitive symptoms in elderly CKD patients [55], while Lu et al. confirmed correlations between nutritional markers and depression in dialysis patients [31]. In our study, the average albumin level among non-dialysis patients was 2.53 ± 0.67 g/dL, which is notably lower than the typical range reported in the general elderly population (3.5–4.5 g/dL) [37,38]. This finding further supports the presence of significant protein-energy depletion in our cohort.

In our study, ALT levels were significantly higher among non-dialysis patients compared to those receiving hemodialysis. This observation may reflect underlying comorbidities, differences in medication exposure, or better-preserved hepatic metabolism in the non-dialysis group. Conversely, low ALT in hemodialysis patients has been described in the literature and may be linked to reduced hepatic perfusion, hemodilution, or protein-energy wasting. Although ALT is not a primary nutritional marker, its difference between groups may provide indirect insights into the complex metabolic changes in CKD patients, and should be further explored in future studies.

Although non-dialysis patients had a higher BMI, they exhibited poorer nutritional markers (i.e., lower albumin levels), which suggests that body weight alone may not reflect true nutritional status. This finding is consistent with the so-called ‘obesity paradox’ described in CKD, where higher BMI may be associated with better survival outcomes but does not necessarily indicate adequate nutritional reserves or metabolic health [56]. Hemodialysis treatment and frequency may positively influence nutritional parameters. Kalender et al. emphasized that treatment modality, depression, malnutrition, and inflammation significantly affect quality of life in CKD patients [19,20,32]. Jiang et al. reported that hemodialysis patients over 65 years of age had lower hemoglobin and albumin levels, both of which are associated with increased mortality [27].

Our results also show that 20.3% of non-dialysis patients were malnourished, compared to only 9.0% of dialysis patients. While dialysis seems to support better nutritional outcomes, the risk of malnutrition remains high in both groups. Sahathevan et al. found that elderly hemodialysis patients had worse nutritional profiles than younger counterparts, while Tsai et al. categorized patients into well-nourished, moderate, and severe malnutrition groups based on PG-SGA scores [33,39]. Cindoğlu and Beyazgül reported a significant relationship between malnutrition and depression in dialysis patients, supporting our findings [45].

Previous studies have extensively examined depression and malnutrition among chronic kidney disease (CKD) patients, especially those undergoing dialysis; however, most did not directly compare dialysis and non-dialysis geriatric populations using standardized tools like the MNA-SF and GDS-SF simultaneously. Wang et al. focused on depression prevalence in elderly CKD patients but did not stratify nutritional and depressive outcomes based on dialysis status [47]. De Alencar et al. explored quality of life and depression among hemodialysis patients without including non-dialysis comparators [46]. Our study adds to the literature by directly comparing these two distinct elderly subpopulations, showing that while malnutrition is significantly more prevalent in non-dialysis patients (20.3% vs. 9.0%), depression scores do not differ significantly, indicating a complex interplay between nutritional and psychological health beyond dialysis status.

Furthermore, while previous research has acknowledged the association between hypoalbuminemia and poor nutritional status or depression individually [31,55], our findings strengthen the evidence by demonstrating a moderate negative correlation between albumin levels and GDS-SF scores, and a positive correlation with MNA-SF scores, highlighting albumin as a dual biomarker of both nutritional and psychological well-being. Unlike earlier studies, we also observed that higher BMI in non-dialysis patients did not correspond with better albumin levels, challenging the assumption that body weight reliably indicates nutritional adequacy in geriatric CKD patients.

Thus, our work contributes novel insights by analyzing both dialysis and non-dialysis elderly CKD groups in parallel, integrating biochemical, nutritional, and psychological parameters, and demonstrating how poor nutrition may coexist with higher BMI and greater depressive symptoms—especially in the non-dialysis geriatric population.

### Limitations

This study has some limitations that should be acknowledged. First, its cross-sectional design prevents the establishment of causal relationships between nutritional status, depression, and dialysis treatment. Longitudinal studies would be more suitable to assess the temporal evolution of these parameters. Second, the sample was limited to patients from a single geographic region in Turkey, which may restrict the generalizability of the findings to other populations. Additionally, the study relied on self-reported tools such as the MNA-SF and GDS-SF, which, although validated, are subject to recall and response biases, particularly in elderly populations with potential cognitive impairments. Laboratory data such as inflammatory markers (e.g., CRP, IL-6), which could have further explained the relationship between malnutrition and depression, were not included in the analysis. Finally, the study did not account for other potential confounders such as socioeconomic status, polypharmacy, dialysis adequacy, medication use, or caregiver support, all of which may influence both nutritional and psychological outcomes in geriatric CKD patients [41,44]. Additionally, our analyses did not include multivariate adjustment for age or other potential confounders, meaning that some differences between dialysis and non-dialysis groups may partly reflect age-related effects. Future studies using regression models are warranted to clarify these relationships. While our cross-sectional findings highlight significant associations between nutritional–metabolic status and depression, they cannot establish predictive value. Future longitudinal studies are required to clarify whether these markers serve as predictors of psychological outcomes in CKD.

## 5. Conclusions

In conclusion, this study highlighted significant associations between nutritional–metabolic status and depressive symptoms in elderly CKD patients, with malnutrition being more frequent among those not receiving hemodialysis. Although higher BMI was observed in this group, lower albumin levels highlight the so-called ‘obesity paradox,’ underscoring that body weight alone is not a reliable marker of nutritional adequacy. Given the baseline differences such as age, BMI, and education, and the absence of multivariate adjustment, the results should be interpreted with caution. Nevertheless, by simultaneously applying standardized tools (MNA-SF and GDS-SF) and incorporating laboratory markers, this study contributes novel insights into the interplay of nutrition and mental health in geriatric CKD. Future longitudinal and multivariate studies are warranted to disentangle causal pathways, explore predictive relationships, and inform integrated screening and intervention strategies aimed at improving quality of life in this vulnerable population.

## Figures and Tables

**Table 1:** Socio-demographic and clinical characteristics of elderly CKD patients by dialysis status (n = 152).

Category	Subgroup	Hemodialysis (n = 78), n (%)	Non-Hemodialysis (n = 74), n (%)	*p*-Value
Gender	Female	40 (51.3%)	42 (56.8%)	0.607
	Male	38 (48.7%)	32 (43.2%)	
Marital Status	Married	58 (74.4%)	55 (74.3%)	1.000
	Single	20 (25.6%)	19 (25.7%)	
	Divorced/Widowed	28 (35.9%)	44 (59.5%)	
Education	Illiterate	32 (41.0%)	27 (36.5%)	0.008
	Primary school	10 (12.8%)	2 (2.7%)	
	Secondary/High school	8 (10.3%)	1 (1.3%)	
	Bachelor’s and above	20 (25.6%)	17 (23.0%)	
Smoking	Yes	58 (74.4%)	57 (77.0%)	0.726
	No	6 (7.7%)	3 (4.1%)	
Alcohol Use	Yes	72 (92.3%)	71 (95.9%)	0.493
	No	2 (2.6%)	2 (2.7%)	
BMI	Underweight	31 (39.7%)	23 (31.1%)	0.001
	Normal weight	30 (38.5%)	27 (36.5%)	
	Overweight	15 (19.2%)	22 (29.7%)	
	Obese	37 (24.4%)		
Hemodialysis Duration	3–11 months	8 (10.3%)		
	1–2 years	18 (23.1%)		
	3–5 years	21 (26.9%)		
	≥6 years	31 (39.7%)		
Weekly Dialysis Sessions	2 times/week	19 (24.4%)		
	3 times/week	59 (75.6%)		

**Table 2:** Comparison of physical and laboratory parameters (mean ± SD).

Variable	Hemodialysis (n = 78), n (%)	Non-Hemodialysis (n = 74), n (%)	*t*-Value	*p*-Value
Age (years)	70.13 ± 7.76	77.07 ± 8.61	−5.222	0.001
Height (cm)	166.83 ± 7.60	164.16 ± 6.52	2.328	0.021
Weight (kg)	69.83 ± 12.05	74.95 ± 11.34	−2.701	0.008
BMI	25.18 ± 4.75	28.44 ± 4.70	−4.252	0.001
Albumin (g/dL)	3.50 ± 0.465	2.53 ± 0.67	9.973	0.001
Hemoglobin (g/dL)	11.16 ± 1.92	12.44 ± 2.03	3.979	0.001
ALT (U/L)	8.53 ± 4.31	17.16 ± 13.06	−5.532	0.001

**Table 3:** Distribution of MNA-SF and GDS-SF scores by groups.

Scale	Hemodialysis (n = 78), n (%)	Non-Hemodialysis (n = 74), n (%)	*t*-Value	*p*-Value
MNA-SF—Normal	51 (65.4%)	38 (51.4%)	2.150	0.033
MNA-SF—At Risk	20 (25.6%)	21 (28.3%)		
MNA-SF—Malnutrition	7 (9.0%)	15 (20.3%)		
GDS-SF—Normal	49 (62.8%)	35 (47.2%)	−1.703	0.091
GDS-SF—Mild Depression	17 (21.8%)	17 (23.0%)		
GDS-SF—Moderate Depression	7 (9.0%)	21 (28.4%)		
GDS-SF—Severe Depression	5 (6.4%)	1 (1.4%)		

**Table 4:** Correlation analysis results for the entire cohort (n = 152).

Variables	Gender (r)	Smoking (r)	Alcohol (r)	HGB (r)	ALB (r)	MNA-SF (r)	GDS-SF (r)
Gender	–						
Smoking	−0.091	–					
Alcohol Use	−0.104	0.377 **	–				
Hemoglobin (HGB)	0.058	−0.236 **	−0.173 *	–			
Albumin (ALB)	−0.165	0.131	0.112	−0.124	–	0.374 **	−0.323 **
MNA-SF	−0.178 *	0.111	0.075	0.078	0.374 **	–	−0.625 **
GDS-SF	−0.209 **	−0.199 *	−0.112	−0.033	−0.323 **	−0.625 **	–

* *p* < 0.05; ** *p* < 0.01, Pearson Correlation.

## Data Availability

The original contributions presented in the study are included in the article; further inquiries can be directed to the corresponding author.

## Abbreviations

ALT	Alanine Aminotransferase
BMI	Body Mass Index
CKD	Chronic Kidney Disease
GDS-SF	Geriatric Depression Scale–Short Form
Hb	Hemoglobin
MNA-SF	Mini Nutritional Assessment–Short Form
